# Anticoagulation and Mortality Rates among Hospitalized Patients with Atrial Fibrillation

**DOI:** 10.1055/s-0038-1626732

**Published:** 2018-01-30

**Authors:** Gregory Piazza, Shelley Hurwitz, Lindsay M. Harrigan, Kathryn L. Jenkins, Benjamin Hohlfelder, John Fanikos, Samuel Z. Goldhaber

**Affiliations:** 1 Cardiovascular Division, Department of Medicine, Brigham and Women's Hospital, Harvard Medical School, Boston, Massachusetts, United States; 2 Center for Clinical Investigation, Brigham and Women's Hospital, Boston, Massachusetts, United States; 3 Department of Pharmacy, Brigham and Women's Hospital, Boston, Massachusetts, United States

**Keywords:** anticoagulation, atrial fibrillation, mortality, adverse events

## Abstract

Atrial fibrillation (AF) is associated with an increased rate of mortality, heart failure, and stroke. We conducted an observational study to assess the relationship between anticoagulation and adverse clinical outcomes in hospitalized patients with AF. We performed a 5,000-consecutive-patient retrospective cohort analysis of anticoagulation prescription and 90-day outcomes in patients with AF hospitalized at Brigham and Women's Hospital from May 2008 to September 2014. All-cause mortality at 90 days was 5.4%. The frequency of death between hospital discharge and day 90 was lower in patients who were anticoagulated at discharge (2.8 vs. 7.1%, *p* < 0.001). Anticoagulation prescription at discharge was associated with a 60% reduction in death between discharge and day 90, after adjustment for confounding factors. Major adverse events at day 90, including death, myocardial infarction, stroke, and major bleeding, were more frequent in patients who were not prescribed anticoagulation at discharge (16.5 vs. 10.4%, *p* < 0.001). In multivariable regression analysis, prescription of anticoagulation at discharge predicted a lower mortality (adjusted odds ratio (OR), 0.4; 95% confidence interval (CI), 0.3–0.53) and lower major adverse event rate (adjusted OR, 0.64; 95% CI, 0.54–0.76) by day 90. In conclusion, all-cause mortality at 90 days was high among inpatients with AF. Patients with AF who were not prescribed anticoagulation at discharge had an increased risk of death at 90 days. Hospitalization represents a special opportunity to optimize cardiovascular risk reduction strategies, including anticoagulation.

## Introduction

In 1998, the Framingham Heart Study reported that atrial fibrillation (AF) had a multivariable-adjusted association with an increased risk of death, 1 and this was subsequently corroborated. 2 3 4 A meta-analysis of 1,009,501 patients, of whom 149,746 had AF, found a 60% increased risk of death in AF, primarily due to increased cardiovascular mortality. 5 A separate meta-analysis of antithrombotic studies showed a 1.6% absolute risk reduction of all-cause mortality in patients with AF who received antithrombotic therapy. 6 While anticoagulation prevents stroke in patients with AF, it may also reduce the risk of other major adverse cardiovascular outcomes. 7 To follow up these previous studies, we assessed the relationship between anticoagulation prescription and fatal and nonfatal cardiovascular outcomes and major adverse clinical events among 5,000 hospitalized patients with AF at Brigham and Women's Hospital (BWH).

## Materials and Methods

### Study Oversight

The study was conducted according to the ethical principles stated in the Declaration of Helsinki. Institutional Review Board approval was obtained. The requirement of informed consent was waived because the study was a quality improvement initiative and a medical record review.

### Study Design

The study was a retrospective cohort analysis using data abstracted through our electronic health record (EHR) at BWH.

### Patient Population

BWH is a 777-bed acute tertiary care facility. Consecutive patients, aged 18 years or older, who were hospitalized at BWH between May 4, 2008, and September 30, 2014, with an admitting diagnosis of AF, were included. For patients with multiple admissions due to AF, only the first admission was included. Patients with valvular heart disease graded “severe” or those with mechanical prosthetic heart valves were excluded.

### Data Query and Collection

Study data were collected by trained research staff (L.M.H., K.L.J., and B.H.) and managed using the Research Electronic Data Capture (REDCap) electronic data capture tool hosted at BWH. 8 REDCap is a secure, web-based application designed to support data capture for research studies.

Patient demographics and baseline clinical characteristics were recorded, including age, gender, race, ethnicity, and comorbid conditions. Clinical characteristics of AF, including symptoms and rhythm management, individual risk factors for stroke and bleeding, and CHA _2_ DS _2_ -VASc 9 and HAS-BLED 10 scores, were obtained from the EHR. Anticoagulation prescription was defined as any order for therapeutic-dose antithrombotic therapy, including non–vitamin K oral anticoagulants, vitamin K antagonists (warfarin), intravenous heparin, low-molecular-weight heparins (LMWH), and fondaparinux. Labile INRs, as a component of the HAS-BLED score, was defined as any clinical mention in the EHR of difficulty maintaining the INR within the target range.

Ninety-day clinical outcomes of acute coronary syndrome, stroke, bleeding events, and all-cause mortality were obtained for all patients by utilizing our EHR, which captures any patient encounter across 6 affiliated hospitals, 17 ambulatory clinics, and numerous private practices. Patient encounters were recorded in the form of discharge summaries, office notes, diagnostic testing reports, medical treatment summaries, and procedure reports, regardless of the facility or office location. Bleeding events were classified according to the International Society of Thrombosis and Haemostasis (ISTH) criteria for major and nonmajor bleeding. 11 The Social Security Death Index (SSDI) was used to identify patients who died during the 90-day follow-up period. Mortality outcomes were confirmed using the Center for Disease Control (CDC) National Death Index (NDI). Causes of death were categorized as cardiovascular or noncardiovascular. The frequency of major adverse events at 90 days, including acute coronary syndrome, stroke, bleed, or death, was recorded. Ninety-day follow-up was completed for 100% of study patients.

### Statistical Analysis

Descriptive statistics, including baseline demographic and clinical characteristics, assessment of stroke and bleeding risk, patterns of stroke prevention in AF, and 90-day clinical outcomes, were stratified as continuous or binary. Continuous variables were assessed for normality of distribution. Normally distributed continuous variables were presented as means with standard deviations. Nonparametric data were presented as median with interquartile ranges. Binary variables were presented as numbers and proportions. Comparative statistics for categorical variables were calculated using the chi-square test or Fisher's exact test. Comparative statistics for continuous variables were calculated using a two-sample *t* -test or Wilcoxon's rank-sum test.

Multivariate regression analyses were conducted to evaluate whether prescription of anticoagulation was associated with death at discharge, death between discharge and day 90, major adverse events at 90 days, and ISTH major bleeding at 90 days, while controlling for several potentially confounding prognostic factors. Variables included in the regression models were selected based on results of univariate analysis and a priori knowledge and were composed of age, gender, CHA _2_ DS _2_ -VASc score, and HAS-BLED score.

All reported *p* -values were two sided. All statistical analyses were performed using SAS version 9.4 (SAS Institute, Cary, North Carolina, United States).

## Results

### Baseline Demographics and Clinical Characteristics

We identified 5,000 unique patients hospitalized at BWH with an admitting diagnosis of AF. The mean age was 69 years ( Table 1 ). The study population was overweight, with a mean body mass index of 29 kg/m ^2^ . Common comorbid conditions included coronary artery disease (22%) and cardiomyopathy (13.6%).

**Table 1:** Baseline demographic and clinical characteristics

Characteristic	*N* = 5,000
Mean age ± standard deviation, y	69.2 ± 13.1
Male, *n* (%)	3,114 (62.3)
Race/ethnicity, *n* (%)	
White	4,394 (87.9)
Black	268 (5.4
Hispanic/Latino	161 (3.2)
Asian	59 (1.2)
Other	14 (0.28)
Cardiomyopathy, *n* (%)	680 (13.6)
Coronary artery disease, *n* (%)	1,113 (22.0)
Prior myocardial infarction or unstable angina	744 (14.9)
Prior coronary intervention	517 (10.3)
Prior coronary artery bypass graft surgery	467 (9.3)
Prior venous thromboembolism, *n* (%)	407 (8.1)
History of falls, *n* (%)	662 (13.2)
Current smoker, *n* (%)	318 (6.4)
Former smoker, *n* (%)	2,188 (43.8)
Prior hospitalization within prior 30 d, *n* (%)	704 (14.1)
Chronic obstructive lung disease, *n* (%)	527 (10.5)
Chronic kidney disease, *n* (%)	683 (13.7)
Hemodialysis, *n* (%)	55 (8.1)

AF was paroxysmal in 40.1%, new in 14.1%, persistent in 10.6%, permanent in 1.4%, and unclassified in 33.8%. Rate and rhythm control were prescribed in 83 and 30%, respectively.

### Risk of Stroke and Bleeding

The median CHA _2_ DS _2_ -VASc score was 3 points ( Table 2 ). The most frequently observed components of the CHA _2_ DS _2_ -VASc score were hypertension (70.4%), age ≥ 75 years (35.9%), and female gender (37.7%). The median HAS-BLED score was 3 points.

**Table 2:** Assessment of stroke and bleeding risk

Characteristic	*N* = 5,000
Heart failure, *n* (%)	955 (19.1)
Hypertension, *n* (%)	3,522 (70.4)
Age ≥ 75 y, *n* (%)	1,796 (35.9)
Age 65–74 y, *n* (%)	1,439 (28.8)
Female, *n* (%)	1,886 (37.7)
Diabetes, *n* (%)	1,045 (20.9)
Prior stroke, transient ischemic attack, or systemic embolism, *n* (%)	881 (17.6)
Vascular disease, *n* (%)	1,641 (32.8)
Renal dysfunction, *n* (%)	325 (6.5)
Liver disease, *n* (%)	227 (4.5)
Prior major bleeding or predisposition to bleeding, *n* (%)	1,512 (30.2)
Labile international normalized ratio, *n* (%)	1,313 (26.3)
Concomitant antiplatelet therapy or nonsteroid anti-inflammatory drugs, *n* (%)	2,581 (51.6)
Alcohol intake ≥ 8 servings per week, *n* (%)	441 (8.8)
Median CHA _2_ DS _2_ -VASc score (interquartile range), points	3 (2–4)
Median HAS-BLED score (interquartile range), points	3 (2–4)

### Clinical Outcomes

**Fig. 1:**
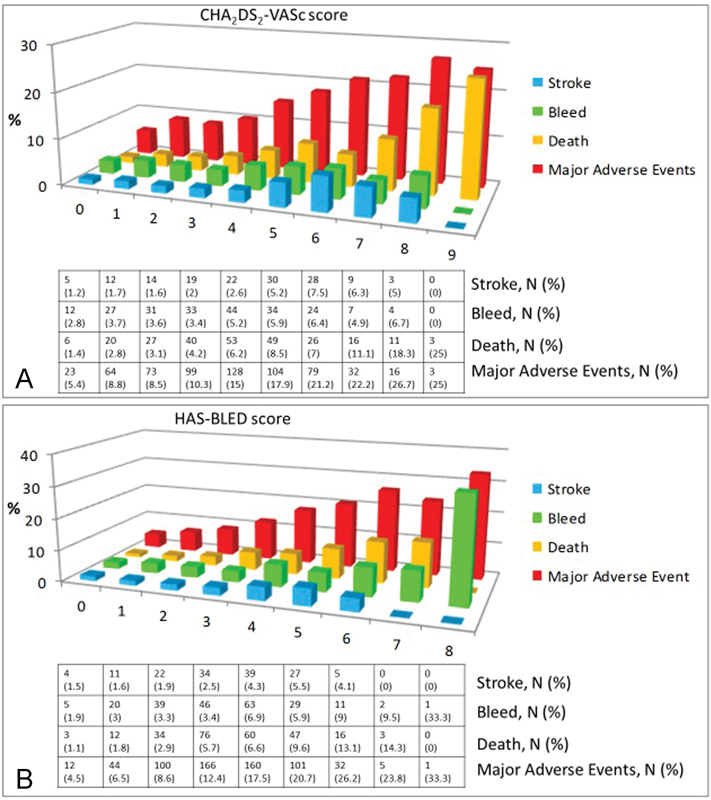
Frequency (%) of adverse events in patients with atrial fibrillation (AF) by CHA _2_ DS _2_ -VASc score ( **A** ). Frequency (%) of adverse events in patients with atrial fibrillation (AF) by HAS-BLED score ( **B** ).

All-cause mortality at 90 days in the overall patient cohort was 5.4%. Cardiovascular causes were noted in 39.2% of inpatient deaths and 12.2% of those taking place between discharge and day 90. Stroke occurred in 2.8%, and bleeding events occurred in 4.3% at 90 days. Ischemic strokes (63.4%) comprised the majority of cerebrovascular events. ISTH major bleeds comprised 42.6% of the bleeding events. Half of the bleeding events were spontaneous. The gastrointestinal tract was the most common site of bleeding (24.1%), followed by surgical (18.1%) and intracranial (6.5%). The overall rate of major adverse events at 90 days was 12.4%, including stroke, MI, bleeding, and death. Major adverse events at 90 days increased with higher CHA _2_ DS _2_ -VASc and HAS-BLED scores ( Fig. 1 ).

### Characteristics and Outcomes of Anticoagulated versus Non-anticoagulated Patients

Hospitalized patients who were prescribed anticoagulation at discharge were slightly younger (mean age: 68.7 vs. 70.1 years, *p* = 0.01). Those with cardiomyopathy (16.4% versus 9%, *p* < 0.001) or a history of heart failure (21.6 vs. 15.2%, *p* < 0.001) were more likely to be anticoagulated. In contrast, those with a history of prior falls (10.2 vs. 18.2%, *p* < 0.001) or dementia (2.3 vs. 5.2%, *p* < 0.001) were less likely to be anticoagulated. The frequency of coronary artery disease was similar between anticoagulated and non-anticoagulated patients (27.9 vs. 30%, *p* = 0.11).

Inpatients who were not prescribed anticoagulation at discharge were more likely to be older, have a prior major bleeding event or predisposition to bleeding, or be prescribed nonsteroidal anti-inflammatory drugs ( Table 3 ). Patients who received inpatient anticoagulation were more likely to be prescribed anticoagulation at discharge compared with those who were not (85.7 vs. 10.4%, *p* < 0.001).

**Table 3:** Assessment of stroke and bleeding risk in patients receiving anticoagulation compared with those who were not

Characteristic	Anticoagulation *N* = 3,105	No anticoagulation *N* = 1,895	*p* -Value
Heart failure, *n* (%)	670 (21.6)	287 (15.2)	<0.001
Hypertension, *n* (%)	2,190 (70.5)	1,338 (70.6)	0.95
Age ≥ 75 y, *n* (%)	1,076 (34.7)	721 (38.1)	0.02
Age 65–74 y, *n* (%)	905 (29.2)	534 (28.2)	0.46
Female, *n* (%)	1,163 (37.5)	1,629 (88.1)	0.62
Diabetes, *n* (%)	663 (21.4)	385 (20.3)	0.38
Prior cerebrovascular accident or systemic embolism, *n* (%)	551 (17.8)	330 (17.5)	0.93
Vascular disease, *n* (%)	995 (32.1)	646 (34.1)	0.14
Renal dysfunction, *n* (%)	403 (13.0)	280 (14.8)	0.49
Liver disease, *n* (%)	108 (3.5)	119 (6.3)	<0.001
Prior major bleed or predisposition to bleeding, *n* (%)	770 (24.8)	742 (39.2)	<0.001
Labile international normalized ratio, *n* (%)	1,037 (33.4)	276 (14.6)	<0.001
Concomitant antiplatelet or nonsteroidal anti-inflammatory drugs, *n* (%)	1,429 (46.0)	1,152 (60.8)	<0.001
Alcohol intake ≥ 8 servings per week, *n* (%)	258 (8.3)	183 (9.7)	0.1
Median CHA _2_ DS _2_ -VASc score (interquartile range), points	3 (2–4)	3 (2–4)	0.92
Median HAS-BLED score (interquartile range), points	3 (2–4)	3 (2–4)	<0.001

The frequencies of inpatient mortality (2.6 vs. 0.03%, *p* < 0.001) and death between hospital discharge and day 90 (7.1 vs. 2.8%, *p* < 0.001) were higher in patients not prescribed anticoagulation at discharge. Major adverse events at day 90, including death, myocardial infarction, stroke, and major bleeding, were more frequent in patients not prescribed anticoagulation at discharge (16.5 vs. 10.4%, *p* < 0.0001). Acute coronary syndromes at day 90 occurred with similar frequency among patients who were prescribed anticoagulation and those who were not (0.9 vs. 1.3%, *p* = 0.13).

In multivariable regression analysis, prescription of anticoagulation at discharge was associated with lower mortality (adjusted odds ratio [OR], 0.4; 95% confidence interval [CI], 0.3–0.53), lower ISTH major bleeding (adjusted OR, 0.5; 95% CI, 0.26–0.81), and a lower major adverse event rate (adjusted OR, 0.64; 95% CI, 0.54–0.76) by day 90. In contrast, increasing CHA _2_ DS _2_ -VASc (adjusted OR, 1.13; 95% CI, 1.01–1.26) and HAS-BLED scores (adjusted OR, 1.16; 95% CI, 1.01–1.32) predicted higher mortality between discharge and day 90. CHA _2_ DS _2_ -VASc (adjusted OR, 1.08; 95% CI, 1.01–1.16) and HAS-BLED scores (adjusted OR, 1.23; 95% CI, 1.13–1.34) also predicted major adverse events by day 90. Neither HAS-BLED score nor prescription of antiplatelet therapy was significantly associated with ISTH major bleeding.

### Evidence-Based Prevention of Stroke

During hospitalization, anticoagulation was prescribed to 57.2% of the patient cohort and to 56.0% with a CHA _2_ DS _2_ -VASc score of at least 1. During hospitalization, warfarin was the most commonly prescribed agent for stroke prevention in AF (55.2%), followed by LMWH (15.7%), unfractionated heparin (13.2%), NOACs (5.8%), and other thromboprophylaxis, including aspirin (11.1%). Anticoagulation was prescribed to 62.1% upon discharge. At discharge, warfarin was the most commonly prescribed agent for stroke prevention in AF (58.7%) followed by LMWH (10.7%), NOACs (6.7%), and other thromboprophylaxis including aspirin (23.9%). Aspirin was prescribed to 17.1% of patients during hospitalization and 12.6% of patients at discharge.

**Fig. 2:**
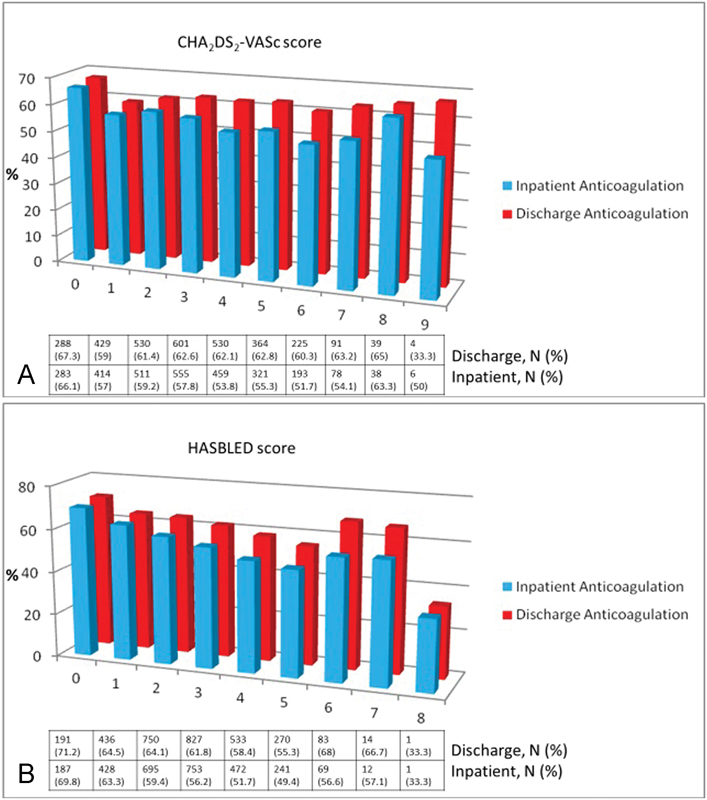
Frequency (%) of anticoagulation prescription during the inpatient stay and at discharge in patients with atrial fibrillation (AF) by CHA _2_ DS _2_ -VASc score ( **A** ). Frequency (%) of anticoagulation prescription during the inpatient stay and at discharge in patients with atrial fibrillation (AF) by HAS-BLED score ( **B** ).

The frequency of anticoagulation prescription remained relatively constant during the inpatient stay and at discharge across CHA _2_ DS _2_ -VASc scores ( Fig. 2A ). Use of anticoagulation decreased with an increasing HAS-BLED score ( Fig. 2B ).

## Discussion

We observed high all-cause mortality at 90 days (5.4%) among hospitalized patients with AF. Cardiovascular causes of death were noted in 39.2% of inpatient deaths and in 12.2% of deaths taking place between discharge and day 90. Anticoagulation prescription at discharge was associated with a 60% reduction in death between discharge and day 90, after adjustment for confounding factors.

Epidemiological cohort studies 2 12 and a systematic analysis of randomized controlled trial data 4 estimate an annual adjusted mortality of 4 to 5% in patients hospitalized with AF. In the Medicare population of patients with AF, annual mortality exceeds 16%. 3 We observed a similarly high mortality in our tertiary care population of patients hospitalized with AF. In our observational study, we also observed that while cardiovascular disease was the most common cause of death, fatal stroke was relatively infrequent. Similarly, in the randomized (rivaroxaban vs. warfarin) ROCKET-AF trial, cardiovascular deaths occurred more than twice as often as strokes. Predictors of higher all-cause mortality included heart failure (hazard ratio, 1.51; 95% CI, 1.33–1.70) and age greater than 75 years (hazard ratio, 1.69; 95% CI, 1.51–1.90). 13 Thus, further advances in anticoagulation strategies may have little effect on improving overall mortality in AF. 14 However, a cardiovascular risk factor management clinic for AF patients has been demonstrated to be clinically effective and cost-saving. 15

Prescription of anticoagulation in AF patients at discharge was associated with a 60% reduction in all-cause mortality between discharge and day 90, even after adjustment for confounding factors. This may have been due, in part, to selection of relatively healthy AF patients. Alternatively, anticoagulation may reduce both cardiovascular and noncardiovascular mortality in patients with AF via effects on other disease processes such as venous thromboembolism.

Our current inpatient study comprised patients with a higher medical acuity compared with our previous outpatient study, 16 with respect to 90-day all-cause mortality (5.4 vs. 1.2%), stroke (2.8 vs. 1.6%), and bleeding events (4.3 vs. 3.7%). The inpatient population in the current study had a higher median HAS-BLED score (3 vs. 2) than our outpatient study. However, the frequency of anticoagulation prescription was higher for hospitalized AF patients at the time of discharge (62.1 vs. 46.9%) compared with the rate in our previously published AF outpatient study. The findings of our study of current hospitalized patients with AF with respect to anticoagulation are consistent with those of the study of the ORBIT-AF Registry on 9,553 outpatients with AF. 17

Anticoagulation prescription rates were low among patients with AF at our tertiary care center. In the international GARFIELD registry of AF patients, prescription rates for anticoagulation in those at high risk for stroke increased to 71% among the final 20% of participants who were enrolled in 2015 and 2016 (presented at the European Society of Cardiology Congress 2017). The findings in our current inpatient study and those in our prior outpatient study 16 are consistent with the low rate of anticoagulation observed during the initial period of enrollment of GARFIELD AF in 2010 (composed entirely of non-U.S. centers). 18 We hope that publication of the current study will provide an educational stimulus to U.S. providers to improve stroke prevention efforts in AF, because we link anticoagulation to prognosis.

There were multiple limitations to this retrospective, observational, administrative dataset. Our electronic data collection did not provide a complete profile of why anticoagulation was omitted in some of the hospitalized patients with AF. The database did not record the reason for hospitalization, which could have influenced prescription of anticoagulant therapy and the observed clinical outcomes. We could not distinguish whether anticoagulation was prescribed specifically for stroke prevention in AF or for some other indication. Our study database did not record data on International Normalized Ratio (INR) values or time within therapeutic range (TTR) for patients prescribed vitamin K antagonists. Despite adjusting for several variables, we may have missed confounding factors that could have impacted clinical outcomes. Cause of death was recorded as cardiovascular and noncardiovascular, and the database did not capture specific cardiovascular causes of death, such as sudden cardiac death. Finally, our study took place at a tertiary care center, and the results might not be representative of the patient populations at other institutions.

Our study provides a “real-world” analysis of the clinical characteristics, stroke and bleeding risks, anticoagulation practices, and clinical outcomes in 5,000 consecutive hospitalized patients with AF. Our analysis is strengthened by having complete (100%) 90-day follow-up for the study cohort.

Recently, there has been increased emphasis on AF as a manifestation of systemic cardiovascular disease. Our study supports the impact of AF on cardiovascular mortality and highlights the magnitude of mortality reduction when AF patients are discharged on anticoagulation.

## Conclusion

Hospitalized patients with AF have high all-cause mortality at 90 days. Anticoagulation prescription at discharge was associated with a 60% reduction in death between discharge and day 90. Hospitalization represents a special opportunity to implement cardiovascular risk reduction strategies, especially anticoagulation.
